# Comparative Diagnostic Accuracy of EUS-Guided Fine-Needle Biopsy Versus Aspiration for Pancreatic Serous Cystic Neoplasms: A Retrospective Cohort Study

**DOI:** 10.3390/jcm15062438

**Published:** 2026-03-22

**Authors:** Alan Chuncharunee, Kazuo Hara, Shin Haba, Takamichi Kuwahara, Nozomi Okuno, Shimpei Matsumoto, Hiroki Koda, Tomoki Ogata

**Affiliations:** 1 Division of Gastroenterology and Hepatology, Department of Medicine, Faculty of Medicine, Ramathibodi Hospital, Mahidol University, Bangkok 10400, Thailand; alan.chu@mahidol.ac.th; 2 Department of Gastroenterology, Aichi Cancer Center Hospital, Nagoya 464-8681, Aichi, Japan; s.haba@aichi-cc.jp (S.H.); kuwa_tak@aichi-cc.jp (T.K.); nokuno@aichi-cc.jp (N.O.); s.matsumoto@aichi-cc.jp (S.M.); h.koda@aichi-cc.jp (H.K.); to.ogata@aichi-cc.jp (T.O.)

**Keywords:** serous cystic neoplasm, pancreatic cystic lesion, endoscopic ultrasound, fine-needle biopsy, fine-needle aspiration, diagnostic yield, Franseen needle

## Abstract

**Background:** Serous cystic neoplasm (SCN) is a common benign pancreatic lesion frequently encountered in practice. However, diagnostic confirmation by Endoscopic ultrasound (EUS)-guided fine needle aspiration (FNA) is often limited by inadequate tissue acquisition. Fine-needle biopsy (FNB) has been increasingly performed. We aimed to compare the diagnostic yield of SCN using FNB and FNA needles and to identify factors associated with successful diagnosis. **Methods**: We retrospectively analyzed 77 patients with pancreatic lesions suspected to be SCN who underwent either EUS-FNB (*n* = 47 procedures) or EUS-FNA (*n* = 50 procedures). The primary outcome was diagnostic yield. Secondary outcomes included predictors of diagnostic yield, which were evaluated using univariate and multivariate logistic regression analyses. Receiver operating characteristic (ROC) analyses were performed to identify the optimal biopsy strategy. **Results**: Diagnostic yield was significantly higher with EUS-FNB than with EUS-FNA (44.68% vs. 14.00%; OR 4.96, 95% CI 1.85–13.28, *p* < 0.01). From univariate and multivariate analysis, larger cyst size, use of the Franseen FNB needle, and a higher number of needle passes were independent factors associated with diagnostic yield. ROC analysis showed modest discrimination for cyst size (AUC 0.69), with an optimal cutoff of ≥17 mm (sensitivity 87.50%, specificity 41.51%). **Conclusions**: EUS-FNB provided superior diagnostic yield compared with EUS-FNA for pancreatic SCN. Lesion size, use of a Franseen needle, and the number of needle passes are key factors associated with successful tissue diagnosis.

## 1. Introduction

SCNs, also known as serous cystadenomas, are common cystic lesions of the pancreas. It has been increasingly detected incidentally with the widespread use of cross-sectional imaging and EUS [1,2]. SCN accounts for 30% of all pancreatic cystic neoplasms. It is predominantly found in women in their sixth decade of life. It is benign in nature with only 0.1% of potential malignancy. Its natural course is usually stable with a growth rate of approximately 6.2% per year and a doubling time of 11.6 years [3]. According to the 2019 WHO classification [4], SCNs are classified into Serous cystadenoma (SCA) and Serous cystadenocarcinoma (SCC). SCA is further subcategorized into five subtypes based on histopathology: microcystic, macrocystic, solid, von Hippel-Lindau-associated (VHL), and mixed serous-neuroendocrine neoplasm (MSNN) subtypes. It is usually managed conservatively with surveillance. Surgical resection is generally reserved for patients with diagnostic uncertainty, symptoms, and excessive growth [5]. According to the American College of Gastroenterology (ACG) guidelines in 2018, there is no need for surveillance once a diagnosis has been made [6]. The classic imaging features of SCN include a microcystic “honeycomb” pattern, multicystic appearance, and the presence of a central stellate scar on EUS or computed tomography (CT) [7]. Unfortunately, these characteristic findings are not always present. Many SCN lesions present with macrocystic, mixed microcystic-macrocystic, or even solid morphology on EUS imaging [8]. As a result, these lesions overlap with other premalignant tumors, including intraductal papillary mucinous neoplasm (IPMN), mucinous cystic neoplasm (MCN), or neuroendocrine tumor (NET) [9,10,11]. This distinction carries important clinical implications. Misclassification of SCN as a mucinous lesion may expose patients to unnecessary surgery and comorbidity. Misclassification in the opposite direction may delay the resection of a premalignant cyst. To differentiate SCN from those aforementioned lesions, cyst fluid sampling is beneficial. EUS-FNA has traditionally been the standard approach for obtaining pancreatic cyst fluid for cytology and biomarker analysis. SCN lesions typically demonstrate low carcinoembryonic antigen (CEA) levels, usually <5 ng/mL. This finding is approximately 95% specific for a nonmucinous cystic neoplasm. In contrast, CEA above 192 ng/mL is highly suggestive of a mucinous lesion with 73% sensitivity and 84% specificity [12]. Values between these two thresholds represent a diagnostic grey zone in which CEA alone cannot reliably differentiate serous from mucinous cysts. Some SCN, particularly the macrocystic type, demonstrate mildly elevated values that overlap with those observed in mucinous cystic lesions [13]. Other biomarkers, such as cyst fluid amylase and glucose, are also not specific enough to serve as definitive diagnostic criteria for SCN. Therefore, cyst fluid analysis alone is often insufficient to confidently establish the diagnosis of SCN [7]. Despite these markers, EUS-FNA cytology for SCN remains unreliable. Adequate numbers of floating serous epithelial cells are often difficult to obtain and preserve. Samples are sometimes compromised by blood contamination from the procedure. These limitations reflect the intrinsic characteristics of SCN, which is typically hypocellular and highly vascularized. As a result, the reported sensitivity of EUS-FNA for diagnosing SCN is low, typically ranging from 13 to 21% [14,15]. These limitations have prompted growing interest in EUS-FNB as a means to improve diagnostic yield. Recent multicenter data suggest that EUS-FNB can achieve a diagnostic yield of up to 87.10% for SCN [16]. The FNB needles, including the Franseen tip and fork-tip designs, incorporate cutting-tip configurations. These features enable core tissue acquisition while preserving histologic architecture. This facilitates pathological assessment of intact cyst wall epithelium and improves diagnostic yield. Nevertheless, evidence remains limited and is derived from small study populations. The optimal needle type and sampling strategy have yet to be established.

In this study, we retrospectively analyzed patients with pancreatic lesions suspected to be SCN on EUS. We compared the diagnostic performance of EUS-FNB with different needle designs to that of conventional EUS-FNA. In addition, we evaluated factors associated with successful tissue diagnosis and identified predictors of diagnostic yield to inform the selection of an optimal tissue acquisition strategy for pancreatic SCN.

## 2. Materials and Methods

### 2.1. Study Design and Setting

This is a single-center retrospective cohort study of patients who underwent EUS-guided tissue acquisition (EUS-TA) for pancreatic lesions suspected to be SCN between July 2004 and July 2025. All procedures were performed at Aichi Cancer Center Hospital, Japan.

### 2.2. Patient Selection

**Figure 1:**
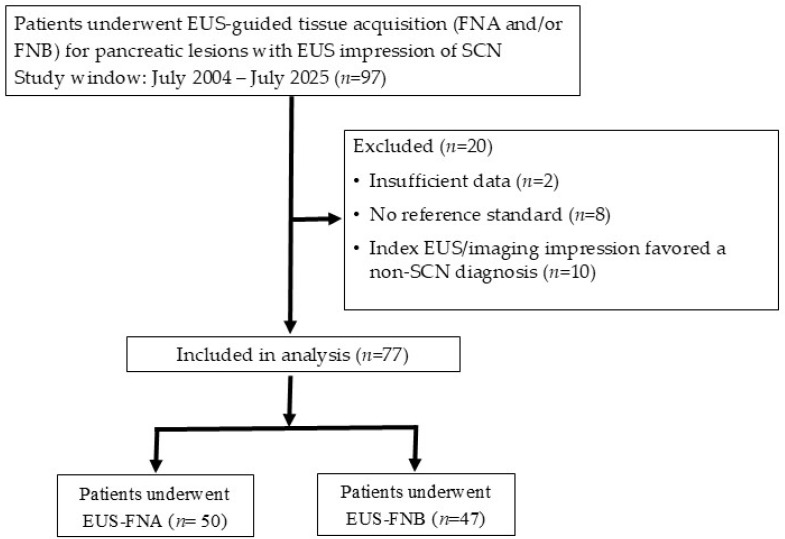
Flowchart of patient selection and inclusion for analysis.

Inclusion criteria were: (1) a pancreatic lesion considered suspicious for SCN on EUS; (2) tissue acquisition performed with either EUS-FNA or EUS-FNB; and (3) availability of a final diagnosis determined using a composite reference standard, defined as either histopathologic confirmation from surgical resection or a presumed diagnosis supported by characteristic imaging findings and stability on clinical and radiologic follow-up for at least 12 months [17]. A lesion was considered suspicious for SCN if one or more of the following EUS features were present: microcystic appearance with multiple small cysts (<1 cm), multicystic morphology without mural nodules, lobulated contour, or the presence of a central stellate scar. In addition, lesions were required to lack the main pancreatic ductal communication and high-risk stigmata for IPMN, such as enhancing mural nodules or significant main pancreatic duct dilatation. Lesions with atypical morphology (macrocystic, mixed, or solid-appearing) were included when clinical context and imaging review by the attending endoscopist supported the possibility of SCN. Specifically, macrocystic variants were suspected when lesions exhibited thin septations without main pancreatic duct communication or worrisome mural nodules. Solid-appearing lesions were suspected when contrast-enhanced EUS showed either a lace-like hypervascular septal enhancement pattern or scattered microcystic foci within an otherwise solid-appearing lesion. Exclusion criteria were: (1) insufficient follow-up or incomplete clinical data, (2) absence of a confirmable reference standard, and (3) index EUS favored a non-SCN diagnosis (Figure 1). The study protocol was approved by the institutional ethics committee (IR071506).

### 2.3. Data Collection

We collected baseline demographic data, lesion characteristics, and procedural details from electronic medical records. With the extensive study duration (2004–2025), needle selection evolved from early cytology-based FNA needles (EZ Shot 3, Olympus America Inc, Center Valley, PA, USA; Expect, Boston Scientific, Marlborough, MA, USA) to modern core-acquisition FNB needles. FNB needles were further grouped by tip design, including Franseen tip (Acquire, Boston Scientific, Marlborough, MA, USA; SonoTip TopGain, Medi-Globe, Achenmuhle, Germany), three-prong asymmetric tip (Trident, Micro-Tech, Nanjing, China), and reverse-bevel tip (Echotip ProCore, Cook Medical, Bloomington, IN, USA). Specimens were processed for cytology and histology. Cytologic and histologic evaluations were performed by dedicated gastrointestinal pathologists according to standard institutional protocols. As this was a retrospective study, formal blinding procedures were not applicable.

### 2.4. Definitions

A diagnostic EUS-FNA result was defined by the presence of glycogen-rich cuboidal epithelial cells with clear cytoplasm arranged in monolayered sheets or cystic structures [14]. A diagnostic EUS-FNB result was defined when paraffin-embedded sections (tissue core and/or cell block) demonstrated characteristic histologic features of SCN. Ancillary testing was performed in cases with an adequate tissue core, defined as at least one evaluable fragment of epithelial cells with preserved cytoarchitecture sufficient for staining. A positive result was defined as MUC-6 or α-inhibin immunoreactivity in cuboidal cells, consistent with serous differentiation. Absence of KRAS mutation (wild-type status) was used as supportive evidence when molecular testing was performed. Ancillary results were integrated into the composite reference standard as supportive evidence when histomorphology was equivocal [18]. Gross morphologic classification was determined based on EUS imaging findings. Lesions were categorized as microcystic, macrocystic, mixed microcystic–macrocystic or solid morphology [19]. The microcystic morphology included the classic honeycomb pattern and the non-uniform microcystic pattern. The classic honeycomb pattern was defined as an aggregation of >6 uniformly sized microcysts arranged in a regular lattice pattern, often with a central stellate scar [19,20]. The non-uniform microcystic pattern was defined as a lesion with multiple small cysts but lacking uniform cyst size, a regular lattice arrangement, or a central scar. Lesions containing both cystic and solid elements were classified according to the predominant architecture. The final diagnosis of SCN was determined using a composite reference standard based on one of the following: (1) surgical histopathology from resected specimens; (2) histologic or cytologic confirmation from EUS-TA demonstrating characteristic features of SCN, with supportive ancillary testing when histologic findings were inconclusive; or (3) a presumed diagnosis based on characteristic imaging features with stability on clinical and radiologic follow-up for at least 12 months [17].

### 2.5. Endpoints

The primary outcomes were the diagnostic yields of EUS-FNB and EUS-FNA. These were defined as the proportion of cases in which EUS-FNB or EUS-FNA resulted in a diagnostic cytologic or histologic result among lesions with a presumed final diagnosis of SCN. Secondary outcomes included identifying factors associated with diagnostic yield and assessing procedural variables that may improve diagnostic success, such as cyst size and the number of needle passes.

### 2.6. Statistical Analysis

Continuous data was reported as mean ± standard deviation or median (interquartile range). Categorical data was reported as counts and percentages. Group comparisons used Student’s *t*-test or Mann–Whitney U test for continuous variables and the χ^2^ or Fisher’s exact test for categorical variables. The primary analysis treated all procedures as independent observations. Logistic regression analysis was used to identify predictors of diagnostic yield. Variables with a *p*-value < 0.10 in the univariate analysis were included in the multivariate model. Variables with established clinical relevance to tissue acquisition, including the number of needle passes and rapid on-site cytology evaluation (ROSE), were included in the multivariate model regardless of univariate statistical significance. Because some patients underwent both EUS-FNA and EUS-FNB, additional sensitivity analyses were performed to address potential non-independence of observations. A generalized estimating equations (GEE) population-averaged model with clustering by patient was used to account for within-patient correlation across procedures. A paired comparison using McNemar’s exact test was also performed for patients who underwent both procedures. An additional separate analysis restricted to independent observations was conducted among patients who underwent only a single procedure. Receiver operating characteristic (ROC) analysis was performed to evaluate the ability of continuous variables to predict diagnostic yield. For variables that remained significant in the multivariate analysis, clinically relevant thresholds for diagnostic yield were determined using Youden’s index (sensitivity + specificity − 1) [21,22]. A two-tailed *p*-value < 0.05 was considered statistically significant. All analyses were conducted using Stata version 18 (StataCorp, College Station, TX, USA).

## 3. Results

### 3.1. Baseline Characteristics

A total of 77 patients were included in the analysis. Among them, 50 patients and 47 patients underwent EUS-FNA and EUS-FNB, respectively. Of these, 20 patients (25.97%) underwent both EUS-FNA and EUS-FNB procedures. There is no significant difference in baseline patient characteristics. The mean age was 61.34 ± 12.70 years. The majority was female (63.64%). The mean cyst size was 23.29 mm (range 6–72 mm). Lesions were commonly located at the pancreatic body or tail in (58.44%). Morphologically, most lesions were microcystic (59.74%), followed by mixed (19.48%), solid (11.69%), and macrocystic (9.09%) variants. However, baseline procedural differences between the EUS-FNA and EUS-FNB groups were identified, reflecting a shift in clinical practice over time. ROSE was available more frequently in the EUS-FNB group than in the EUS-FNA group (70.21% vs. 42.00%, *p* = 0.01). The use of suction in the procedure is higher in EUS-FNB than in EUS-FNA, but not statistically significant (68.09% vs. 52.00%, *p* = 0.10). The median number of passes was 2 (IQR, 2–3) with no difference between the two groups (*p* = 0.46). In terms of needle selection, the 22G needle was the most common size at 58.44%. No significant difference in needle size was noted between groups (*p* = 0.67). There was an increasing use of FNB needles in the later years of the study period, reflecting evolving clinical practice. Among the EUS-FNB cohort, FNB needle tip configurations included the Franseen tip (61.70%), the three-prong asymmetric tip (17.02%), and the reverse-bevel tip (14.89%). The median follow-up period was 32 months (Table 1). Cyst fluid carcinoembryonic antigen (CEA) analysis was performed in 16 cases, and all resulted in less than 192 ng/mL.

### 3.2. Diagnostic Yield of EUS-FNA Versus EUS-FNB

Among the patients who underwent EUS-FNA, a diagnostic yield was obtained in 7 of 50 cases (14.00%; 95% CI: 7.00–26.20%). In contrast, EUS-FNB achieved diagnostic yield in 21 of 47 cases (44.68%; 95% CI: 31.41–58.76%). EUS-FNB was associated with a significantly higher diagnostic yield compared with EUS-FNA (OR 4.96, 95% CI 1.85–13.28, *p* < 0.01). Using GEE to account for within-patient correlation, EUS-FNB was associated with a significantly higher diagnostic yield compared with EUS-FNA (OR 4.90, 95% CI 2.10–11.40, *p* < 0.01). A sensitivity analysis restricted to independent observations (*n* = 57 patients who underwent a single procedure) showed a similar result. Diagnostic yield remained significantly higher with EUS-FNB compared with EUS-FNA (44.44% [12/27] vs. 13.33% [4/30], Fisher’s exact *p* = 0.01). Among the 20 patients who underwent both EUS-FNA and EUS-FNB, 6 discordant pairs were identified. All favored EUS-FNB, with none favoring EUS-FNA. The exact McNemar test was statistically significant (*p* = 0.03). Ancillary diagnostic testing was available in 21 cases. These ancillary findings were interpreted together with cytopathologic features and imaging findings to support the diagnosis. Among patients with microcystic morphology, EUS-FNB provided a tissue diagnosis in 15 of 29 patients (51.72%). Diagnostic yields were 40.00%, 50.00%, and 20.00% in macrocystic, mixed, and solid-appearing lesions, respectively. Regarding safety, there were no major complications, such as pancreatitis, perforation, or severe hemorrhage, in either group. No minor adverse events, including cyst infection or severe abdominal pain requiring medical intervention or prolonged hospitalization, were reported.

### 3.3. Factors Associated with Diagnostic Yield: Univariate Analysis

In univariate logistic regression, larger cyst size was associated with a higher diagnostic performance (OR 1.07 per mm, 95% CI 1.02–1.11, *p* < 0.01). Franseen tip FNB needles also demonstrated significantly higher odds of diagnostic yield than conventional FNA needles (OR 4.16, 95% CI 1.25–13.80, *p* = 0.02). The three-prong asymmetric tip FNB needles demonstrated a trend toward higher diagnostic yield compared to the FNA needles (OR 4.16, 95% CI 0.99–17.59, *p* = 0.05). The effect of reverse-bevel needles could not be estimated because all cases achieved a diagnostic outcome. Needle size, number of needle passes, lesion location, ROSE, and suction were not significantly associated with diagnostic efficacy.

### 3.4. Independent Predictors of Diagnostic Yield: Multivariate Analysis

In the multivariate model, cyst size (OR 1.06 per mm, 95% CI 1.01–1.12, *p* = 0.02), the use of Franseen tip FNB needles (OR 4.34, 95% CI 1.10–17.17, *p* = 0.04), and number of passes (OR 2.06 per pass, 95% CI 1.10–3.88, *p* = 0.03) remained independently associated with diagnostic yield. In contrast, three-prong asymmetric tip needles were not independently associated with diagnostic yield after adjustment (OR 4.61, 95% CI 0.89–23.81, *p* = 0.07). ROSE was also not associated with diagnostic yield (*p* = 0.66) (Table 2).

### 3.5. Receiver Operating Characteristic Analysis

**Figure 2:**
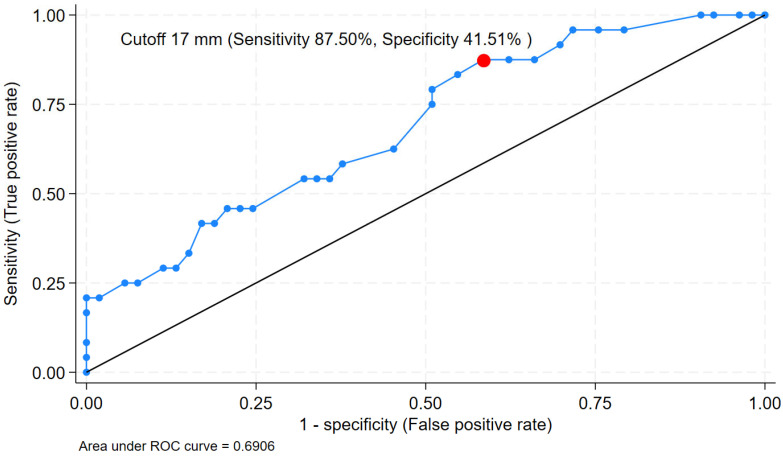
ROC curve (blue line) for cyst size in predicting diagnostic yield. The AUC was 0.69 (95% CI 0.56–0.82). The optimal cutoff ≥ 17 mm (red dot) demonstrated high sensitivity (87.50%) with limited specificity (41.51%). The black diagonal line indicates the line of no discrimination. This suggests that lesions at or above this threshold are most likely to yield a diagnosis with EUS-FNB. The modest discriminative ability suggests that size alone should serve as a supportive guide.

**Figure 3:**
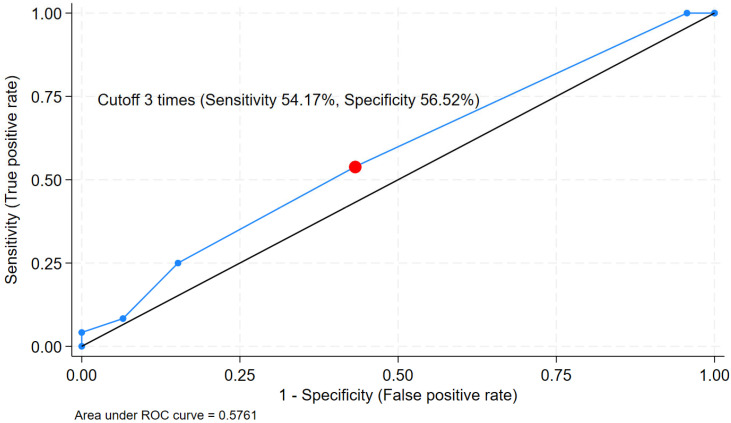
ROC curve (blue line) for the number of passes in predicting diagnostic yield. The AUC was 0.58 (95% CI 0.44–0.71). The optimum cutoff ≥ 3 passes (red dot) demonstrated moderate sensitivity (54.17%) and specificity (56.52%). The black diagonal line indicates the line of no discrimination. This suggests that three or more passes may improve the likelihood of diagnosis. The low discriminative ability indicates that the number of passes is a poor independent predictor of diagnostic yield.

The ROC analysis for cyst size showed modest discriminative ability (AUC 0.69, 95% CI 0.56–0.82). An optimal cutoff of ≥17 mm yielded a high sensitivity of 87.50% but a limited specificity of 41.51% (Figure 2). In contrast, the number of needle passes demonstrated poor discriminative ability (AUC 0.58, 95% CI 0.44–0.71) (Figure 3). An empirical cutoff of ≥3 passes showed a sensitivity of 54.17% and a specificity of 56.52%, indicating that the number of passes was not a robust independent predictor of diagnostic success.

## 4. Discussion

Our study demonstrated that EUS-FNB achieved a significantly higher diagnostic yield than EUS-FNA in suspected SCN lesions. These findings are consistent with previous reports [15,16]. However, our diagnostic yield of 51.72% with the EUS-FNB in microcystic lesions was lower than the 87.09% reported by Kim et al. [13]. Although baseline characteristics and cyst location were similar, the patient selection criteria and needle designs likely account for the difference. The previous study achieved a high pretest probability by recruiting exclusively classic honeycombing morphology. Our microcystic subtype included both classic honeycomb and non-uniform microcystic patterns. Lesions lacking a honeycomb arrangement may have less organized epithelial architecture or more intervening stroma. These may reduce the likelihood of obtaining representative serous epithelium. While the previous study exclusively used the cutting-tip needles (Franseen tip and fork-tip), our study included a broader range of needle designs. In our cohort, the Franseen tip and three-prong asymmetric tip needles accounted for approximately two-thirds of the total EUS-FNB cases.

In our study, the Franseen tip and the three-prong asymmetric tip FNB needle designs significantly outperformed the FNA needle. Although the three-prong asymmetric tip design showed a significant association with diagnostic performance in univariate analysis, this association did not remain significant after multivariate adjustment. This finding likely reflects limited statistical power and potential overlap with other factors. Overall, these findings support the Franseen tip needle as the most reliable option. Despite the modest diagnostic yield of EUS-FNB in our study, it represents a clinically meaningful fourfold improvement over EUS-FNA. This improvement is particularly relevant in lesions with non-classic morphology, where imaging alone may not be sufficient to confidently exclude malignancy. In such situations, histopathologic confirmation from EUS-FNB can increase diagnostic certainty and guide management. This was reflected in the 21 patients who achieved a definitive tissue diagnosis with EUS-FNB. All were managed non-surgically and discharged from intensive surveillance. This highlights the practical role of tissue diagnosis in reducing unnecessary surveillance and surgery in accordance with current guideline recommendations [6]. Failed EUS-FNA sampling may lead to repeat procedures, prolonged surveillance, or surgery. This places an additional burden on healthcare resources. Procedural adjuncts such as ROSE have been used to improve diagnostic yield, particularly for solid pancreatic masses [23]. However, its routine use is limited by the need for an on-site cytopathologist [24]. In our study, ROSE was not associated with diagnostic yield. This may be due to the limited cellularity of SCN lesions.

ROC analysis identified a cyst size cutoff of ≥17 mm as the optimal threshold for predicting diagnostic yield. The high sensitivity (87.50%) suggests that most lesions at or above this size are likely to acquire adequate tissue for diagnosis, although specificity was limited. For lesions <17 mm, the likelihood of obtaining diagnostic yield appears lower. Surveillance may be reasonable when imaging features are typical for SCN. For lesions ≥17 mm with diagnostic uncertainty, EUS-FNB may be considered. However, the AUC for cyst size (0.69) indicates only modest discriminatory ability, suggesting that cyst size alone should not be used as a strict decision threshold. The number of passes showed poor discriminatory performance on ROC analysis (AUC 0.58) despite remaining an independent predictor of diagnostic yield in multivariate analysis. This difference likely reflects the distinct purposes of the two analytic approaches. Logistic regression identifies independent association after adjustment for other variables, while ROC analysis evaluates the ability of a single continuous variable to discriminate outcomes across thresholds. In practice, performing additional passes may incrementally increase the probability of achieving diagnostic yield when combined with appropriate needle selection and adequate lesion size. However, the number of passes alone is insufficient as a standalone decision criterion. Based on these findings, EUS-FNB using a Franseen tip needle with at least three passes may be considered in lesions ≥ 17 mm with diagnostic uncertainty, although further validation is needed. In clinical practice, this cutoff should support decision-making rather than be treated as a strict rule. The decision to perform EUS-FNB should be discussed among other alternative strategies, including continued surveillance or surgical resection.

This study has several strengths. First, it represents one of the largest cohorts focused on SCN. The relatively long median follow-up of 32 months further supports diagnostic confirmation. Second, our study explicitly reflects real-world situations. Unlike prior studies that relied primarily on surgically resected specimens, we used a composite diagnostic approach. The diagnosis was based on imaging findings, histopathology or cytopathology results from EUS-TA, along with ancillary testing. This approach better reflects how SCN is diagnosed and managed in routine practice, where most patients are treated non-surgically. Our cohort also included a broad spectrum of lesion morphologies and needle designs. This allows evaluation of factors associated with diagnostic yield. Despite the limited number of cases in some subgroups, we were able to demonstrate the independent predictive value of the Franseen tip FNB needle. Third, we performed multiple confirmatory statistical analyses to strengthen the validity of the primary comparison of diagnostic yield between EUS-FNB and EUS-FNA. Finally, we explored the cutoff values using the ROC analysis. By applying Youden’s method, we identified cyst size and number of passes that balance sensitivity and specificity of diagnostic yield. Although the AUC values were modest, these findings may still provide useful guidance when considering tissue acquisition.

This study has several limitations. First, the retrospective single-center design may introduce selection and referral bias. Needle selection and procedural techniques were determined by the operating endoscopist. In addition, pathologists had access to clinical information during routine sample evaluations, which may have introduced interpretation bias. Although consecutive patients were included using consistent inclusion criteria, residual confounding cannot be entirely excluded. Secondly, although the number of patients included in this study was sufficient to reach a conclusion on the primary outcome comparing the diagnostic yield of EUS-FNB and EUS-FNA, the sample size may have been insufficient for more detailed subgroup or multivariable analyses. A post hoc analysis of primary comparison indicated that the study had 92.55% power (α = 0.05) to detect the observed difference. Regarding the multivariable model, the number of events was small relative to the number of predictors. Five predictors were included with 28 diagnostic events, resulting in an events-per-variable ratio of 5.6. It is below the conventional threshold of 10. This raises the possibility of model overfitting and unstable estimated effect sizes. For example, odds ratios could not be estimated for the reverse-bevel tip FNB needle group because all cases in this subgroup achieved a diagnostic result. Although the result appears favorable, the small number of cases in this subgroup limits the reliable estimation of the effect size and warrants cautious interpretation. Therefore, variables were selected not only based on univariate significance (*p* < 0.10), but also on clinical relevance. Third, the study was conducted at a high-volume referral cancer center. This may limit the generalizability of the findings to community settings with lower EUS-TA volumes or without dedicated GI pathologists. Fourth, procedural heterogeneity may have partly influenced the results. The long study period may have introduced variation related to changes in EUS equipment, needle design, and pathology processing over time. Conventional FNA needles were used more frequently in the earlier years of the study, whereas the dedicated cutting-tip FNB needles were gradually adopted later. This temporal shift may have introduced confounding between needle type and procedural era. Baseline procedural differences were also observed between the two groups, including higher rates of ROSE and suction in the EUS-FNB group. These differences may confound the comparison between EUS-FNB and EUS-FNA. To account for this, needle type, ROSE, and use of suction were included as covariates in the multivariable analysis to assess their association with diagnostic yield. Stratification by time period was considered but not performed because it would have substantially reduced statistical power within already small subgroups. Fifth, a definitive pathological gold standard was not available for all cases because most patients were managed non-surgically. As a result, resected specimens for histopathologic confirmation were limited. Therefore, conventional measures of diagnostic performance, including sensitivity, specificity, and accuracy, could not be calculated due to verification bias. To address this limitation, the final diagnosis was based on a composite reference standard. Lastly, there might be a violation of the assumption of independent observations at the procedure level because 20 patients underwent both EUS-FNA and EUS-FNB. To address this issue, several sensitivity analyses were conducted. The primary approach uses GEE models to account for clustering. This method accounts for within-patient correlation without discarding data. Additional analyses included a paired comparison using McNemar’s exact test for the patients who underwent both procedures. Another separate analysis was restricted to independent observations. These results were consistent with the primary findings. This suggests that the differences in diagnostic yield are more likely related to procedural factors rather than patient-level factors.

Our study provides real-world evidence supporting the superiority of EUS-FNB over EUS-FNA for tissue diagnosis in SCN. With improved diagnostic yield, patients can avoid repeated imaging and unnecessary surgery. This may also reduce unnecessary procedures and improve healthcare resource utilization. Future studies should evaluate the cost-effectiveness of EUS-FNB by comparing its procedural and hospitalization costs with the cumulative costs of long-term imaging surveillance. They should also identify the economic threshold at which early tissue diagnosis becomes more cost-effective than continued surveillance.

## 5. Conclusions

Obtaining an accurate tissue diagnosis for SCN remains challenging yet clinically crucial. This study showed that EUS-FNB achieved a higher diagnostic yield than EUS-FNA, with the Franseen tip needle showing the best performance. Exploratory analyses suggested that larger cyst size and multiple needle passes may increase diagnostic success, although these findings require further validation. When tissue confirmation is required for lesions with diagnostic uncertainty, EUS-FNB may improve diagnostic yield and support more appropriate patient management.

## Figures and Tables

**Table 1:** Baseline patient, cyst, and procedural characteristics.

	EUS-FNA (*n* = 50)	EUS-FNB (*n* = 47)
**Patient characteristics**		
**Age, mean ± SD (years)**	61.06 ± 12.84	62.36 ± 13.41
**Female sex, *n* (%)**	31 (62.00)	31 (66.00)
**Cyst size, mean (range), mm**	22.50 (6–72)	25.26 (8–72)
**Location, *n* (%):**		
Head	18 (36.00)	16 (34.04)
Neck	4 (8.00)	3 (6.38)
Body	20 (40.00)	18 (38.30)
Tail	8 (16.00)	10 (21.28)
**Morphology, *n* (%):**		
Microcystic	27 (54.00)	29 (61.70)
Macrocystic	4 (8.00)	5 (10.64)
Solid	6 (12.00)	5 (10.64)
Mixed	13 (26.00)	8 (17.02)
**Procedural characteristics**		
**Needle size, *n* (%):**		
19G	19 (38.00)	13 (27.66)
22G	20 (40.00)	33 (70.21)
25G	4 (8.00)	1 (2.1)
**No. of passes, median (IQR)**	3 (2–3)	2 (2–3)
**ROSE performed, *n* (%)**	21 (42.00)	33 (70.21)
**Suction applied, *n* (%)**	26 (52.00)	32 (68.09)
**Follow-up duration, median (months)**	40 (20–69)	25 (14–51)

Abbreviations: EUS, endoscopic ultrasound; FNA, fine-needle aspiration; FNB, fine-needle biopsy; SD, standard deviation; IQR, interquartile range; ROSE, rapid on-site evaluation; G, gauge.

**Table 2:** Predictors of diagnostic yield for SCN (univariate and multivariate logistic regression).

Variable	Univariate OR (95% CI)	*p*-Value	Multivariate OR (95% CI)	*p*-Value
**Cyst size (per mm)**	1.07 (1.02–1.11)	< 0.01	1.06 (1.01–1.12)	0.02
**Needle type**				
Conventional FNA (ref)	1.00	-	-	-
Franseen tip FNB	4.16 (1.25–13.80)	0.02	4.34 (1.10–17.17)	0.04
Three-prong asymmetric tip FNB	4.16 (0.99–17.59)	0.05	4.61 (0.89–23.81)	0.07
Reverse-bevel tip FNB	Not estimable		Not estimable	
**Needle size**				
25G (ref)	1		-	-
22G	3.33 (0.36–30.95)	0.29	-	-
19G	2.08 (0.19–22.67)	0.55	-	-
**No. of passes (per time)**	1.35 (0.87–2.13)	0.18	2.06 (1.10–3.88)	0.03
**ROSE performed**	1.70 (0.82–3.54)	0.15	1.25 (0.47–3.36)	0.66
**Suction applied**	0.86 (0.31–2.43)	0.78	-	-

Abbreviations: OR, odds ratio; CI, confidence interval; FNA, fine-needle aspiration; FNB, fine-needle biopsy; ROSE, rapid on-site cytologic evaluation; ref, reference category. ORs are reported per unit increase for continuous variables (per mm for cyst size and per pass for number of passes).

## Data Availability

No new data were created or analyzed in this study.
